# Cell surface Nucleolin represents a novel cellular target for neuroblastoma therapy

**DOI:** 10.1186/s13046-021-01993-9

**Published:** 2021-06-02

**Authors:** Chiara Brignole, Veronica Bensa, Nuno A. Fonseca, Genny Del Zotto, Silvia Bruno, Ana F. Cruz, Fabiana Malaguti, Barbara Carlini, Fabio Morandi, Enzo Calarco, Patrizia Perri, Vera Moura, Laura Emionite, Michele Cilli, Francesco De Leonardis, Annalisa Tondo, Loredana Amoroso, Massimo Conte, Alberto Garaventa, Angela R. Sementa, Maria V. Corrias, Mirco Ponzoni, Joao N. Moreira, Fabio Pastorino

**Affiliations:** 1 Laboratory of Experimental Therapies in Oncology, IRCCS Istituto Giannina Gaslini, Genoa, Italy; 2 CNC – Center for Neurosciences and Cell Biology, Center for Innovative Biomedicine and Biotechnology (CIBB), University of Coimbra, Faculty of Medicine (Polo 1), Coimbra, Portugal; 3 TREAT U, SA - Parque Industrial de Taveiro, Lote 44, 3045-508 Coimbra, Portugal; 4 Department of Research and Diagnostics, IRCCS Istituto Giannina Gaslini, Genoa, Italy; 5 Department of Experimental Medicine, University of Genoa, Genoa, Italy; 6 UC – University of Coimbra, CIBB, Faculty of Pharmacy, Pólo das Ciências da Saúde, Azinhaga de Santa Comba, 3000-548 Coimbra, Portugal; 7 Department of Pathology, Istituto Giannina Gaslini, Genoa, Italy; 8 Stem Cell Laboratory and Cell Therapy Center, IRCCS Istituto Giannina Gaslini, Genoa, Italy; 9 Animal Facility, IRCSS Ospedale Policlinico San Martino, Genoa, Italy; 10 Department of Pediatric Oncology, Azienda Ospedale Policlinico di Bari, Bari, Italy; 11 UOC Oncologia Pediatrica, Ospedale Meyer, Florence, Italy; 12 UOC Oncologia, IRCCS Istituto Gaslini, Genoa, Italy

**Keywords:** Neuroblastoma, Cell surface protein, Nucleolin, Targeted therapy, Nanotechnology

## Abstract

**Background:**

Neuroblastoma (NB) represents the most frequent and aggressive form of extracranial solid tumor of infants. Nucleolin (NCL) is a protein overexpressed and partially localized on the cell surface of tumor cells of adult cancers. Little is known about NCL and pediatric tumors and nothing is reported about cell surface NCL and NB.

**Methods:**

NB cell lines, Schwannian stroma-poor NB tumors and bone marrow (BM)-infiltrating NB cells were evaluated for the expression of cell surface NCL by Flow Cytometry, Imaging Flow Cytometry and Immunohistochemistry analyses. The cytotoxic activity of doxorubicin (DXR)-loaded nanocarriers decorated with the NCL-recognizing F3 peptide (T-DXR) was evaluated in terms of inhibition of NB cell proliferation and induction of cell death in vitro, whereas metastatic and orthotopic animal models of NB were used to examine their in vivo anti-tumor potential.

**Results:**

NB cell lines, NB tumor cells (including patient-derived and Patient-Derived Xenografts-PDX) and 70% of BM-infiltrating NB cells show cell surface NCL expression. NCL staining was evident on both tumor and endothelial tumor cells in NB xenografts. F3 peptide-targeted nanoparticles, co-localizing with cell surface NCL, strongly associates with NB cells showing selective tumor cell internalization. T-DXR result significantly more effective, in terms of inhibition of cell proliferation and reduction of cell viability in vitro, and in terms of delay of tumor growth in all NB animal model tested, when compared to both control mice and those treated with the untargeted formulation.

**Conclusions:**

Our findings demonstrate that NCL could represent an innovative therapeutic cellular target for NB.

**Supplementary Information:**

The online version contains supplementary material available at 10.1186/s13046-021-01993-9.

## Background

Neuroblastoma (NB) represents the most frequent and aggressive form of extra-cranial solid tumor of infants, responsible for 15% of childhood cancer deaths [1]. For high-risk NB patients, chemotherapy and radiotherapy remain the main first-line treatment choices, which nevertheless lead to remarkable toxicity, development of drug resistance, and in 50% of cases to recurrence and progression [2]. Thus, novel therapeutic strategies are urgently needed. On the other hand, targeted therapies are often limited by the lack of specific receptors. This, together with their often limited tumour penetration, represent major drawbacks related to the shortage of novel cancer therapies in the clinic.

Nucleolin (NCL) is one of the most abundant proteins of the nucleolus, involved in DNA and RNA metabolism, ribosome biogenesis, ribosome(r) RNA synthesis and processing, chromatin organization and stability, cytokinesis, cell proliferation, angiogenesis, apoptosis regulation, stress response and microRNA processing [3–5]. Recently, it has been demonstrated to be also implicated in pathological conditions, especially in tumorigenesis and viral infection, rendering NCL a potential target for the development of anti-tumor and anti-viral strategies [4, 5]. Up-regulated NCL, observed in several solid and blood cancers, might contribute to tumorigenesis by increasing rRNA synthesis and the assembly of functional ribosomes. Interestingly, NCL is not located only in the nucleolus, but also in the cytoplasm and on the cell membrane. Enhanced NCL membrane expression has recently been reported on activated lymphocytes, angiogenic endothelial cells and several types of cancer cells, acting as a binding partner of different molecules implicated in cell differentiation, adhesion, leukocyte trafficking, inflammation, angiogenesis, lymphangiogenesis and tumorigenesis [6, 7]. For all these reasons, NCL might represent a potential biomarker for cancer diagnosis and a target for cancer treatment.

Christian S. et al. in 2003 demonstrated that NCL is expressed at the cell surface of tumor and endothelial cells in angiogenic tumor blood vessels [8]. Moreover, they showed that the F3 tumor-homing peptide (KDEPQRRSARLSAKPAPPKPEPKPKKAPAKK) is able to specifically recognize and, therefore, to associate to and to internalize into cell surface NCL-expressing tumor cells [8]. More recently, the cytotoxic potential of a F3 peptide-targeted, doxorubicin (DXR)-entrapping, pH-sensitive liposomal formulation was demonstrated on cell surface NCL-expressing breast and glioblastoma cancer cells [9, 10]. Recently, NCL was reported to be a functional binding protein for salinomycin in NB stem cells [11] but nothing has been reported about cell surface NCL and NB. In this manuscript, cell surface NCL is proposed as a new targetable protein for NB in pre-clinical models, and it is suggested as a possible future NB marker and an innovative therapeutic cellular target in the clinical setting. The cytotoxic activity of the GMP-grade NCL-recognizing, DXR-loaded, pH sensitive liposomal formulation PEGASEMP™ [9] was here tested against NCL-positive NB cells, in vitro and in biologically relevant murine models of human NB. We demonstrated that our specific drug delivery approach based on the recognition of the cell surface NCL allowed significant improvements in therapeutic efficacy compared to the administration of DXR encapsulated in untargeted nanoparticles. Together with the validation of cell surface NCL expression on Schwannian stroma-poor NB tumors and on bone marrow-infiltrating NB cells, this leads to hypothesize a plausible use of this “targeted nanodrug” in patients suffering from NB.

## Materials and methods

### Cells lines, animal models and human samples

Human (IMR-32, HTLA-230, SH-SY5Y and SK-N-AS) and murine (NXS2) neuroblastoma (NB) cell lines were grown in complete Dulbecco’s Modified Eagle Medium (DMEM) medium, as previously described [12–15]. In some experiments, IMR-32 cells were infected with retrovirus expressing the firefly luciferase (luc) gene, as previously reported [13]. Luciferase activity of IMR-32-luc cells was confirmed by bio-luminescent imaging (BLI, Lumina-II, Caliper Life Sciences, Hopkinton, MA) after a 10 min incubation with 150 μg/mL d-luciferin (Caliper Life Sciences) diluted in cell culture medium, as previously described [13–15]. Normal human dermal fibroblasts (FIBRO) were purchased from American Type Culture Collection (ATCC, Manassas, VA); human skin keratinocytes cells (HaCaT) were kindly provided by Dr. Flavio Curnis (San Raffaele Scientific Institute, Italy); both cell lines were grown in complete RPMI-1640 medium, as previously described [12]. Cells were tested for mycoplasma contamination, characterized by cell proliferation and morphology evaluation, and authenticated at time of experimentation by multiplex STR-profiling test (PowerPlex® Fusion, Promega, Milan, Italy) by BMR Genomics (Padova, Italy) and validated using ATCC STR, DSMZ STR and NCBI databases.

Female athymic Nude-Foxn1^*nu*^ (*nu/nu*) mice (Envigo, Bresso, Italy) were housed under pathogen-free conditions. In accordance with the 3Rs policy, experiments were reviewed and approved by the licensing and ethical committee of Ospedale Policlinico San Martino and by the Italian Ministry of Health (n. 661/2016-PR).

For the pseudo-metastatic model, HTLA-230 cell line (4 × 10^6^ cells in 200 μL culture medium) was inoculated in the tail vein of four-week-old *nu/nu* mice, as described [12, 15, 16]. For the orthotopic model, IMR-32 (wild-type and luc-transfected) or SH-SY5Y cell lines (1 × 10^6^ cells in 10 μL culture medium) were inoculated in the left adrenal gland of five-week-old *nu/nu* mice, as described [12, 15, 17]. No mice died as a result of the surgery. Mice body weight and general physical status were daily recorded. When any sign of discomfort or poor health arose (i.e., abdominal dilatation, dehydration, paraplegia, > 20% weight loss) mice were anaesthetized with xilezine (Xilor 2%, Bio98 Srl, Milan, Italy) and sacrificed by CO_2_ inhalation. The day of euthanasia was recorded as the day of death.

Samples derived from Schwannian-stroma poor, poorly differentiated, NB primary tumors at relapse (Patient Codes: Fig. 2a) and Bone Marrow (BM)-infiltrating NB cells from patients at either relapse (*n* = 7) or onset (*n* = 29) (Patient Codes: Table 1S) were provided by BIT (Integrated Tumor Bio-Bank of Gaslini Institute, Tissue Section), Istituto G. Gaslini, Genoa, Italy. Collection and manipulation of human samples were approved by the competent Ethics Committee and informed consent was obtained from each patient in accordance with the Declaration of Helsinki.

### In vitro and in vivo NCL expression

The expression of cell surface NCL was evaluated on i) NB cell lines, both in culture and after being injected in mice either intravenously or orthotopically in the adrenal gland, ii) tissue sections of Schwannian stroma-poor NB primary tumors and iii) bone marrow (BM)-infiltrating NB cells. Specifically, Flow Cytometry (FC, Becton-Dickinson Immunocytometry Systems, Gallios Flow Cytometer, Beckman Coulter), Imaging Flow Cytometry (IFC, Image StreamX II, Luminex) and Immunohistochemistry (IHC) analyses were performed.

The expression of surface NCL on NB cell lines, on healthy cells (fibroblasts and keratinocytes), on cells derived from Schwannian stroma-poor NB primary tumors and on BM-infiltrating NB cells were processed and evaluated by FC. Specifically, 5 × 10^5^ NB or healthy cells were incubated for 25 min (min) at 4 °C with anti-NCL (mouse IgG1 moAb, AlexaFluor488, clone 364–5, Abcam) monoclonal antibody (moAb). In case of tumor cells, fragments were mechanically dissociated and sequentially filtered through 100 and 70 μm cell strainers to obtain a single cell suspension. BM-infiltrating NB cells, after red cells lysing, were processed as cultured cells.

For multiple staining, cells (1 × 10^5^ cells/tube) were incubated with the following moAbs: anti-CD45 (PE-Cy7, clone 2D1, eBioscience™), anti-CD56 (APC, clone CMSSB, eBioscience™), anti-NCL (same as above), anti-GD_2_ (PE, clone 14G2a, BioLegend) and anti-B7-H3 (PE, clone DCN.70, BioLegend). CD45 is a marker of hematological cells; CD56, GD_2_ and B7-H3 are markers of NB cells [18–20]. After being washed with PBS (2 mM EDTA, 1% FBS), all cells were analyzed by FC.

For IFC analyses, cultured NB cells were detached by the use of 5 mM EDTA in PBS, to thwart cell clusters formation. Cells (6 × 10^5^/tube) were then incubated with the IgG1 moAb anti-NCL AlexaFluor488 for 25 min at 4 °C) and, after being washed (2 mM EDTA, 1% FBS in PBS), incubated with an AlexaFluor488-conjugated secondary moAb (anti-IgG1 secondary moAb, 20 min at 4 °C) to amplify the mean fluorescence intensity (MFI) of the positive population. Control cells were incubated with the AlexaFluor-conjugated anti-IgG1 secondary moAb alone. To check for possible artifacts, each sample was acquired through both a cell sorter (FACSAria III) and an IFC. For IHC analyses, the fully automated Immunostainer BenchMark® ULTRA Roche from Ventana Medical Systems was used. Paraffin-embedded tumor tissues sections (3 μm), derived from NB cell lines injected in mice, were de-paraffinized and incubated with anti-NCL moAb ([EPR7952] ab129200, abcam) diluted 1:750 in Dako Real™ Antibody Diluent (Dako). Sections from healthy murine kidney and adrenal gland were stained as non-tumoral tissues. The ultraView Universal DAB detection kit from Ventana was used to detect the binding of primary antibody. Sections were counterstained with Hematoxylin II (Ventana).

### In vitro binding and internalization of NCL-recognizing nanocarriers

Rhodamine-labeled, untargeted and F3 peptide (recognizing NCL ([9, 21])-targeted, pegylated pH-sensitive (DOPE:CHEMS:HSPC:CHOL:DSPE-PEG:DSPE-PEG-MAL, 4:2:2:2:0.18:0.12 M ratio) liposomes (NT-Rhoda and T-Rhoda, respectively), were prepared and characterized as described [9]. Five × 10^5^ NB cells were incubated with 200 mM total lipid of each tested nanocarrier for 30 min at 4 °C and for 1 h at 37 °C, for binding and uptake study, respectively. Samples were then washed with PBS (2 mM EDTA, 1% FBS), and Rhoda fluorescence associated with NB cells was evaluated by FC. Results are either presented by histograms or expressed as Mean Ratio Fluorescence Intensity (MRFI) normalized over control cells (without liposomes incubation). The co-localization of NCL and T-Rhoda was evaluated by IFC. Six × 10^5^ NB cells were co-incubated with anti-NCL AlexaFluor-488-conjugated moAb and T-Rhoda for 25 min at 4 °C. Cells were then washed, as already described, and analyzed by IFC.

The specific internalization of T-Rhoda into NB cell lines was also evaluated by confocal microscopy. Five × 10^4^ NB cells were incubated for 1 h at 37 °C. After washing and cytospin centrifugation, samples were fixed for 30 min with 4% paraformaldehyde in PBS, incubated for 40 min in PBS containing 2% BSA and then stained with anti-N-CAM moAb (mouse anti-CD56, clone 123C3, Invitrogen) to reveal plasma membrane localization. Binding of the primary moAb was detected with FITC-conjugated goat anti-mouse IgG (Invitrogen); cell nuclei were counterstained with 4′ 6-diamidino-2-phenylindole (DAPI) (Thermo Scientific). The cellular distribution of liposomal nanocarriers (red), N-CAM (green) and DAPI (blue) fluorescence was analyzed using a laser scanning spectral confocal microscope (TCS SP2-AOBS; Leica Microsystems, Heidelberg, Germany). The experiment was repeated 5 times.

### In vitro cytotoxicity of NCL-recognizing, doxorubicin-loaded nanocarriers

#### Cell proliferation assay

Untargeted and targeted, doxorubicin (DXR)-loaded, pH-sensitive liposomes (NT-DXR and T-DXR, respectively) were prepared and characterized, as described [9]. NB cell lines were labeled with Carboxyfluorescein diacetate succinimidyl ester (CFSE, CellTrace™ CFSE Cell Proliferation Kit, Thermo Fisher Scientific), according to manufacturer’s instruction and seeded in 12-well plates (7.6 × 10^4^ and 6.4 × 10^4^, depending on the cell line). The day after seeding, cells were treated for 30 min with NT-DXR and T-DXR (0.5 and 1 μM DXR), in duplicate for each experimental condition. Then, cells were recovered with fresh, complete medium. After additional 95 h and 30 min, cells were collected and processed for FC analysis. The results are expressed as mean fluorescence intensity (MFI) of CFSE; due to the dilution of the dye during rounds of division, cells whose proliferation has been inhibited, presented a higher MFI with respect to cells that went through a physiological proliferation.

#### Viability assay

NB cell lines and skin keratinocytes cells were seeded in 96-well plates (4.6–6.8 × 10^4^ cells/well, depending on the cell line, and 2.5 × 10^4^ cells/well, respectively). The day after seeding, cells were treated with NT-DXR and T-DXR (0.1–2 μM DXR), in quadruplicate for each experimental condition, for 30 min, as described before. After additional 95 h and 30 min, the cells were processed to assess cell viability using the tetrazolium salt assay (MTS, CellTiter 96® Aqueous One Solution Cell Proliferation Assay, Promega).

### In vivo therapy and toxicity

In the pseudo-metastatic model, 1 h after free doxorubicin-resistant [16] HTLA-230 cell inoculation, mice were randomly assigned to different groups (*n* = 16 mice/group) and intravenously (*i.v).* treated with 3.5 mg/kg NT-DXR or T-DXR, twice a week, for 4 weeks. In the orthotopic model, 14 days after IMR-32-luc cell inoculation, mice were randomly assigned to different groups after BLI evaluation (*n* = 6 and *n* = 8 mice/group, for imaging and survival experiments, respectively) and *i.v.* treated for imaging as above, and with 3.5 mg/kg of free-DXR, NT-DXR or T-DXR, twice a week, for 4 weeks, for survival experiments. In all the in vivo experiments, control mice (CTR) received HEPES-buffered saline. BLI monitoring and survival times were used as the main criterion for determining treatment efficacy. Mice were weighed 24 h after each treatment. Moreover, in the systemic chronic toxicity experiment, mice were anesthetized with xylazine 24 h after the last day of treatment and blood was collected, as previously reported [15, 22–25]. Hematological levels of red blood cells (RBC), hemoglobin (HGB), hematocrit (HCT), reticulocytes (RET), platelets (PLT) and white blood cells (WBC) as well as clinical chemistry levels of serum albumin (ALB), phosphatase alkaline (ALP), glutamic-pyruvic transaminase (ALT), glutamic oxaloacetic transaminase (AST), lactate dehydrogenase (LDH), creatine phosphokinase (CK) and creatinine (CREA) and urea were quantified. All the reported evaluations were performed at the Mouse Clinic, IRCCS Ospedale San Raffaele (Milano).

### Statistics

The analyses were performed with Prism 5 software (GraphPad, La Jolla, CA): one- and two-way analyses of variance (ANOVA) with Tukey’s Multiple Comparison Test were used to evaluate differences within treatments; survival curves were drawn as Kaplan-Meier Cumulative Proportion Surviving graphs, and corresponding *p*-values were calculated by the use of the log-rank (Mantel-Cox) test. Asterisks indicate the following p-value ranges: * = *p* < 0.05, ** = *p* < 0.01, *** = *p* < 0.001.

## Results

### Cell surface NCL is expressed on neuroblastoma cell lines, on neuroblastoma xenografts and on patient-derived neuroblastoma tumors and BM-infiltrating neuroblastoma cells

**Fig. 1:**
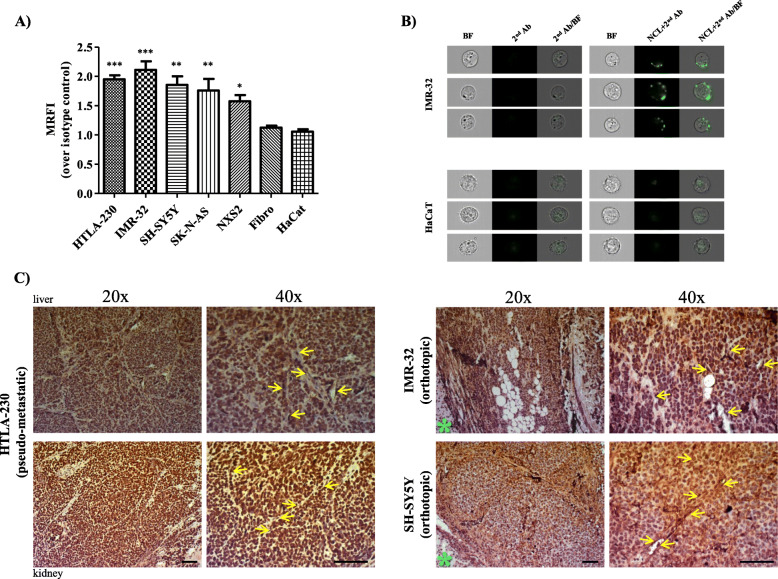
NCL is a cell surface protein in NB cells in vitro and ex-vivo. **A** Flow Cytometry (FC) analyses of the constitutive expression of NCL in human (HTLA-230, IMR-32, SH-SY5Y,SK-N-AS) and murine (NXS2) NB cell lines and in humanFibroblasts (Fibro) and keratinocytes (HaCaT). Cells were washed with PBS (2 mM EDTA, 1% FBS) and incubated with anti-NCL AlexaFluor488 moAb (NCL-A488; 5 μg/mL in 100 μL). NCL-positive cells were counted by FC. NCL-positive cells are expressed as mean relative fluorescence intensity (MRFI) normalized over cell staining with isotype-matched (mouse IgG1) AlexaFluor-488 conjugated Ab. Columns (MRFI ± S.D.). ***, *p* < 0.001: HTLA-230 and IMR-32 vs Fibro and HaCat; **, *p* < 0.01: SH-SY5Y and SK-N-AS vs Fibro and HaCat; *, *p* < 0.05: NXS2 vs Fibro and HaCat. **B** Representative images of surface expression of NCL in single IMR-32 and HaCaT cells, processed and incubated as described above, and counted by Imaging Flow Cytometry (IFC). A secondary moAb was used to amplify the mean fluorescence intensity of the NCL-positive population. BF: Bright Field; 2nd Ab: AlexaFluor 488-conjugated secondary moAb; NCL: anti-NCL AlexaFluor488 moAb (green). **C** Immunohistochemistry (IHC) staining on formalin-fixed tumor sections, either in the liver (upper panels) or kidney (lower panels), following injection in nude mice of 4 × 10^6^ HTLA-230 cells (through the tail vein of mice; pseudo-metastatic model), or 1 × 10^6^ of SH-SY5Y or IMR-32 cells (injected in the left adrenal gland of mice; orthotopic model). Tumors were harvested following 35 (SH-SY5Y), 40 (HTLA-230) or 42 (IMR-32) days of cancer cell lines inoculation. 20x and 40x: degree of magnification. Bar: 100 μm. Brown: NCL expression. Yellow arrows: NCL-positive, tumor endothelial cells. Green asterisks: healthy kidney

**Fig. 2:**
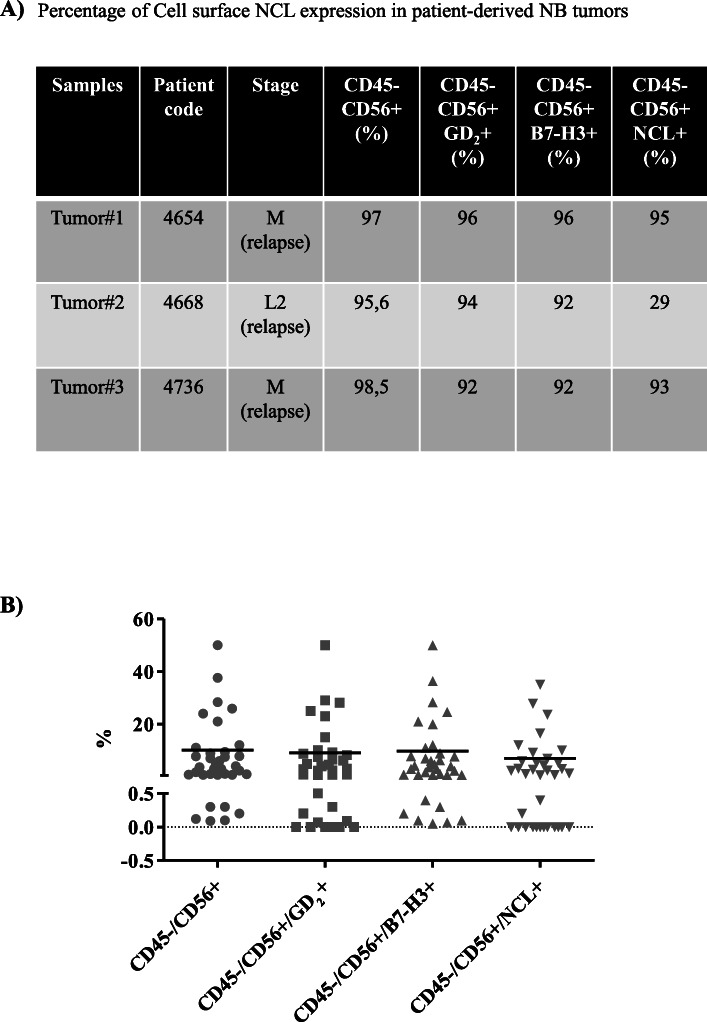
Percentage of cell surface NCL expression in NB tumors and in BM-infiltrating NB cells. Single cell suspension from patient-derived tumors (**A**) and BM-infiltrating NB cells (**B**) were incubated for 25 mi at 4 °C with anti-CD45, anti-CD56, anti-GD_2_, anti-B7-H3 and anti-NCL moAbs, and analyzed by FC. Bars in scatter plots represent mean percentage (%) of positivity

Constitutive expression of cell surface NCL was evaluated in NB cell lines by FC and IFC analyses. Fibroblasts and skin keratinocytes were used as controls. FC analyses demonstrate that NCL is significantly expressed, at different extent, on the cell surface of all NB cells analyzed (Fig. 1a), while IFC analyses confirm cell surface localization of NCL (Figs. 1b and 1S). NCL expression on healthy cells results negligible, indicating that cell surface NCL expression is tumor specific in vitro (Fig. 1a-b). Furthermore, NCL expression was evaluated on formalin-fixed tumor sections derived from HTLA-230, IMR-32 and SH-SY5Y human NB cell lines injection in nude mice. IHC evaluations display a marked NCL positivity in each well-established NB animal model, both on tumor and tumor endothelial cells (Fig. 1c). Importantly, on healthy kidney and adrenal gland from the same mice, NCL expression results very low and localized only at the nucleus level, indicating that cell surface NCL is tumor specific also in vivo (Figure 2S)*.* To clinically validate the expression of NCL on human NB, immunophenotype staining of patient-derived primary tumor cells and BM-infiltrating NB cells, firstly validated by cytomorphology evaluation, was carried out. FC analyses performed on mechanically dissociated primary tumor cells demonstrate that the NB cells, expressing the CD56, GD_2_ and B7-H3 NB-specific markers, also express cell surface NCL (Fig. 2a). Furthermore, 67% (24/36) of BM-infiltrating NB cells, derived from patients at either relapse or onset, express cell surface NCL (Fig. 2b).

Altogether, these results demonstrate that cell surface NCL is expressed by NB cells, representing a potential marker and a targetable antigen for NB tumors, and supporting the rationale for pre-clinical evaluation of cell surface NCL-targeted therapy in NB.

### F3 peptide-targeted nanocarriers bind and internalize into NB cells in vitro

**Fig. 3:**
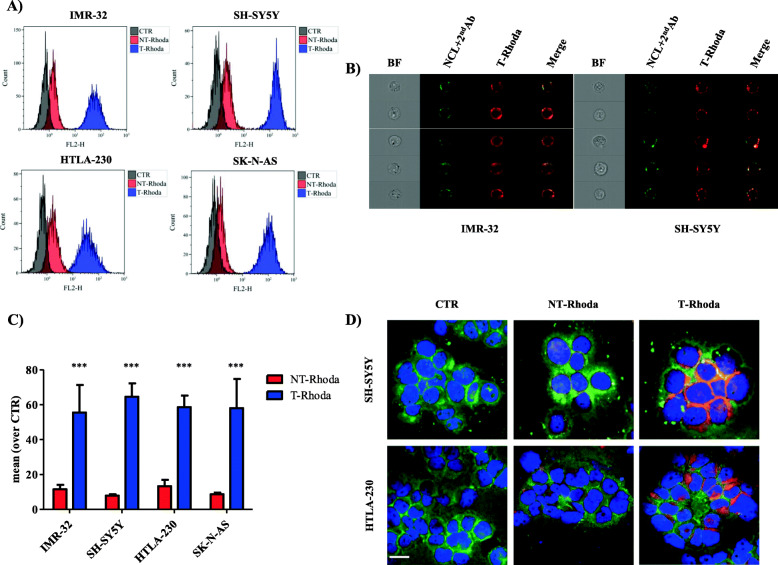
Liposomes functionalized with NCL-recognizing F3 peptide specifically bind and internalize into NB cells in vitro. **A** Binding to NB cell lines. Cells were incubated for 30 min at 4 °C with NT-Rhoda and T-Rhoda. Rhoda fluorescence associated with NB cells was evaluated by FC. CTR: NB cells only. **B** Co-localization of NCL and T-Rhoda. IMR-32 and SH-SY5Y cells were co-incubated with anti-NCL-A488 and T-Rhoda and analyzed by Imaging Flow Cytometry (IFC). BF: Bright Field; NCL: anti-NCL AlexaFluor488 moAb (green); 2nd Ab: AlexaFluor 488-conjugated secondary moAb; T-Rhoda: F3-nanocarrier (Red); Merge: co-localization (yellow). **C** Uptake by NB cell lines. Cells were incubated for 1 h at 37 °C with NT-Rhoda and T-Rhoda. Rhoda fluorescence associated with NB cells was evaluated by FC. Results are expressed as MRFI normalized over control cells. Columns: MRFI ± S.D. ***, *p* < 0.001: T-Rhoda vs NT-Rhoda. **D** Specific internalization into NB cell lines. SH-SY5Y and HTLA-230 cells were incubated as reported in C. After washing, samples were fixed and incubated with an anti-NCAM monoclonal antibody for plasma membrane localization. Green: N-CAM; Red: nanocarriers-Rhoda; Blue: DAPI. Bar: 30 μm

The NCL-recognizing F3 peptide was coupled at the extremity of poly (ethylene glycol)-grafted, pH-sensitive, rhodamine stained liposomes (T-Rhoda), as described [9]. The capability to specifically bind to NB cells was first assessed at 4 °C by FC and IFC analyses, using the untargeted liposome as control (NT-Rhoda). Figure 3a shows that T-Rhoda is more efficient than NT-Rhoda in binding to all tested NB cell lines. The specific recognition of cell surface NCL by T-Rhoda was demonstrated in IFC analyses, where the co-localization (Merge, yellow) of NCL staining (green) and T-Rhoda (red) binding is shown (Fig. 3b). The absence of co-localization of NCL and NT-Rhoda (Figure 3S) confirm the specificity of recognition and binding of T-Rhoda to cell surface NCL.

Then, to assess the cell internalization properties of T-Rhoda, NB cell lines were incubated at 37 °C with T- and NT-Rhoda, as control. FC analyses show a selective NB cells uptake of T-Rhoda, significantly higher than that obtained with NT-Rhoda (Fig. 3c). Confocal analyses confirm this cellular association specificity. In fact, to reveal plasma membrane localization, NB cell lines treated with T-Rhoda (red) were co-incubated with a moAb specific for the cellular adhesion molecule N-CAM (green). As shown in Fig. 3d, T-Rhoda associate and is internalized more efficiently than NT-Rhoda.

### NCL-recognizing, DXR-loaded pegylated and pH-sensitive nanoparticles inhibit NB cell proliferation and reduce NB cell viability in vitro

**Fig. 4:**
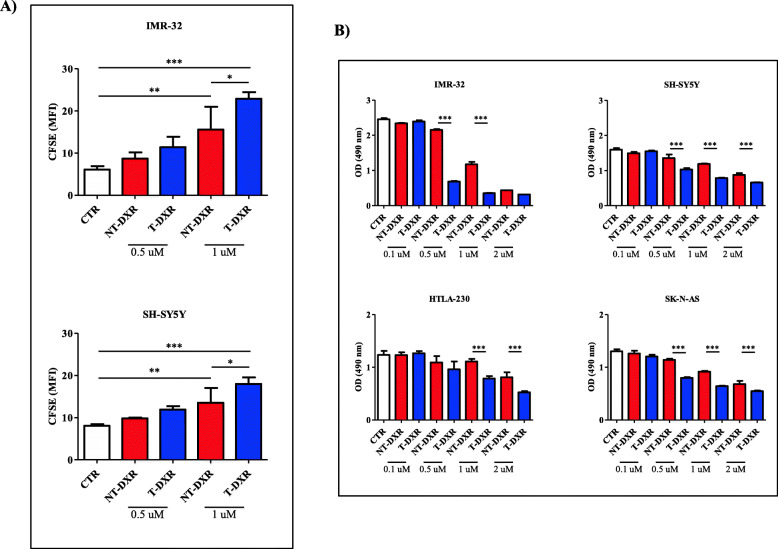
In vitro effects of NCL-recognizing F3 peptide-targeted, doxorubicin (DXR)-loaded, pH-sensitive pegylated nanoparticles on NB cell proliferation and viability. **A** CFSE assay. IMR-32 and SH-SY5Y NB cell lines were treated with 0.5 and 1 μM of both NT-DXR and T-DXR. At 96 h after of treatment, cells were collected and processed by FC to detect CFSE fluorescence. Results are expressed as MFI. Columns: MFI ± S.D. *, *p* < 0.05, T-DXR 1 μM DXR vs NT-DXR 1 μM DXR; **, *p* < 0.01 NT-DXR 1 μM DXR vs CTR; ***, *p* < 0.001, T-DXR 1 μM DXR vs CTR. **B** Viability assay: NB cells were treated with NT-DXR and T-DXR (0.1–2 μM DXR) and processed to determine cytotoxicity through the MTS assay. Results are expressed as optical density (OD) determined at 490 nm. Columns: OD ± S.D. ***, *p* < 0.001:T-DXR vs NT-DXR

The cell proliferation inhibition and cytotoxic effects of doxorubicin (DXR), encapsulated into the NCL-recognizing (T-DXR) and the non-targeted (NT-DXR) liposomes were first evaluated in vitro*.* CFSE-stained NB cell lines were treated with 0.5 and 1 μM DXR encapsulated in T-DXR and NT-DXR, and the inhibition of cell proliferation was assessed by FC. Figure 4a shows that NT-DXR and T-DXR impaired NB cells proliferation in a dose-dependent manner, with T-DXR being more potent than NT-DXR, compared to untreated cells (CTR) already at the lower dose of DXR used. The more potent therapeutic effect of T-DXR compared to that obtained by NT-DXR becomes significant at the highest drug concentration used (T-DXR vs CTR, ***: *p* < 0.001; NT-DXR vs CTR, **: *p* < 0.01; T-DXR vs NT-DXR, *: *p* < 0.05) (Fig. 4a).

Cytotoxicity experiments performed by treating NB cells with different concentrations of DXR-containing NT and T show that T-DXR is significantly more effective than NT-DXR in inducing a dose-dependent reduction of NB cell viability (Fig. 4b). Finally, T-DXR resulted more efficacious to inhibit cell proliferation (Figure 4SA) and to reduce cell viability (Figure 4SB) in those cells expressing significantly higher amount of cell surface NCL (Fig. 1a), providing the evidence that NCL mediates the therapeutic effects of T-DXR on cell proliferation and survival.

### T-DXR delay tumor growth in metastatic and orthotopic NB animal models

**Fig. 5:**
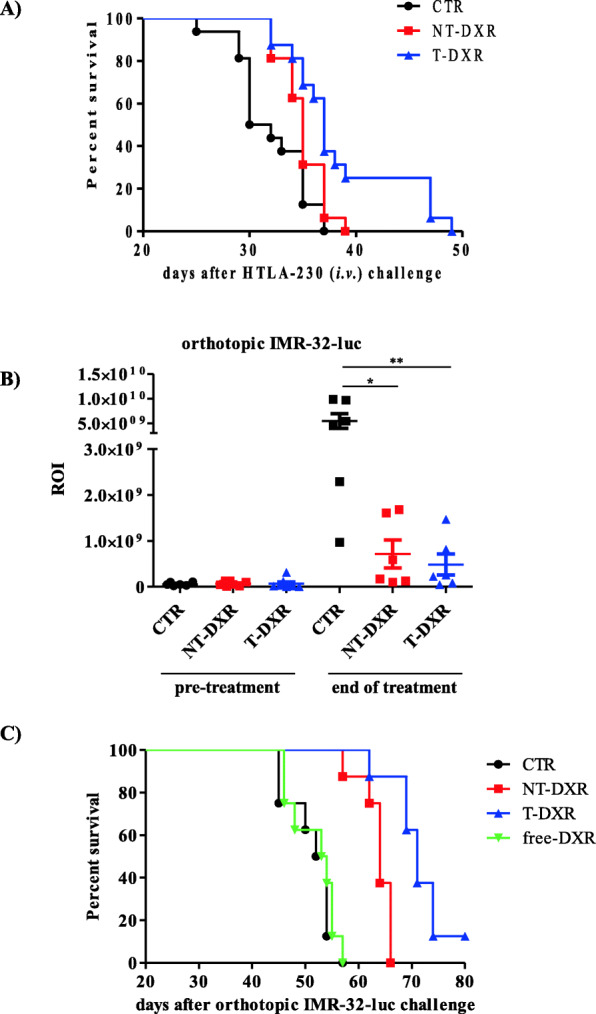
Anti-tumor effects of NCL-recognizing F3 peptide-targeted, doxorubicin (DXR)-loaded, pH-sensitive pegylated liposomes, in a pseudo-metastatic and in a orthotopic murine model of neuroblastoma. **A** Survival in the pseudo-metastatic model. Mice injected in the tail vein with HTLA-230 NB cells were treated with 3.5 mg/kg of not targeted (NT-DXR) or F3 targeted liposomes (T-DXR), twice a week, for four weeks. Control mice (CTR) received HEPES-buffered saline. Statistics: NT-DXR vs CTR, *p* = 0.0323; T-DXR vs CTR, *p* = 0.0003; T-DXR vs NT-DXR, *p* = 0.0189. **B** Tumor growth delay and (**C**) survival in the orthotopic model. Mice, injected in the adrenal gland with 1 × 10^6^ luciferase-transfected IMR-32 (IMR-32-luc) NB cells, were treated for imaging as above, and with 3.5 mg/kg of free-DXR, NT-DXR or T-DXR, twice a week, for four weeks, for survival experiments. In (**B**) tumor growth of IMR-32-Luc was monitored by BLI. Photon counts in the tumor Region of Interest (ROI) are reported at pre-treatment and 24 h post the end of treatment. Results on dot-plots are presented ± SD. *, *p* < 0.05 and **, *p* < 0.01: NT-DXR and T-DXR vs CTR, respectively. In (**C**) NT-DXR vs CTR and vs free-DXR, *p* = 0.0001; T-DXR vs CTR and vs free-DXR, *p* < 0.0001; T-DXR vs NT-DXR, *p* = 0.0015

The anti-tumor efficacy of T-DXR was evaluated in pseudo-metastatic and in orthotopic animal models of NB, aiming at recapitulating conditions observed in relapsed/refractory NB patients. The results obtained in the pseudo-metastatic model indicate that T-DXR can specifically kill NB cells in vivo, leading to a significant increase of life span, relative to NT-DXR-treated or untreated mice (NT-DXR vs CTR, *p* = 0.0323; T-DXR vs CTR, *p* = 0.0003; T-DXR vs NT-DXR, *p* = 0.0189) (Fig. 5a). Since NCL is expressed on both tumor and endothelial tumor cells (Fig. 1c) the anti-tumor potential of T-DXR was also evaluated on a well-established and well-vascularized orthotopic NB animal model [14]. Doses and schedules of treatment used were the same as for the pseudo-metastatic model. In a first experiment, tumor growth and response to treatments of luciferase-transfected NB cells were monitored by BLI. Photon counts in the tumor Region of Interest (ROI) before and 24 h post the end of treatment (day 14 and 43 from cells injection, respectively) show that T-DXR led to a superior delay in tumor growth compared to that enabled by NT-DXR (T-DXR vs CTR, **: *p* < 0.01; NT-DXR vs CTR, *: *p* < 0.05) (Fig. 5b). Importantly, no weight loss was evidenced in any of the treated groups (Fig. 5S). Moreover, no hematological and non-hematological toxicities emerged 24 h post the end of treatment (Figure 6S and 7S). In the second experiment, orthotopically implanted NB-bearing mice were used to verify the anti-tumor efficacy of T-DXR in terms of increased animal life span. Compared to CTR mice and those treated with free DXR, both NT-DXR and T-DXR treatments delay tumor growth (NT-DXR vs CTR and vs free-DXR, *p* = 0.0001; T-DXR vs CTR and vs free-DXR, *p* < 0.0001); however, T-DXR efficacy was significantly more pronounced than that of NT-DXR (T-DXR vs NT-DXR, *p* = 0.0015) (Fig. 5c), suggesting that the specific targeting of cell surface NCL is essential to achieving a stronger anti-tumor effect also in vivo.

## Discussion

In this manuscript, we demonstrate that the protein Nucleolin is expressed on the cell surface of neuroblastoma (NB) cells. This expression, firstly validated in human NB cell lines in culture, is maintained when the same cells are inoculated into clinically relevant NB murine models that recapitulate tumor circulating cells, tumor growth and metastatic spreading conditions observed in relapsed/refractory NB patients [12–17].

Of importance is the fact that the expression of cell surface NCL was demonstrated on NB cells derived from patients suffering with metastatic bone marrow tumor infiltration, both at recurrence and at onset, and on NB primary tumors masses, supporting NCL as a new marker and an useful cellular target molecule also in the clinical setting.

The GMP grade, DXR-loaded, pegylated liposomal PEGASEMP™, a pH-sensitive liposome formulation able to specifically recognize and kill cell surface NCL-expressing breast cancer cells [9, 21] was herein tested as a proof-of-concept device as druggable cellular target also in pre-clinical NB setting. PEGASEMP™ can inhibit NB cell proliferation and reduce NB cell viability in vitro, and can delay tumor growth in two different animal models of NB. Altogether, these results demonstrate that cell surface NCL might be used for the selective targeting of NB cells, through intravenous administration of nanoparticles encapsulating not only chemotherapeutic agents, but also retinoids or macromolecules like siRNA or miRNA mimics molecules, specific for NB oncogene silencing or for tumor suppressor miRNA replacement, respectively, as previously demonstrated [15, 23, 26].

Cell surface NCL has been shown to be expressed on endothelial cells in angiogenic tumor blood vessels [8], making NCL a useful marker for simultaneous targeting of cancer cells and tumor endothelium [21]. Herein, we confirmed the expression of NCL in the endothelium of both metastatic and primary NB tumors, therefore suggesting the dual cellular targeting capability of nanoparticles functionalized with the F3 peptide.

NCL expression on NB cells infiltrating the bone marrow show that about 70% of the analyzed patients express NCL on the external surface. This percentage is slightly lower than that of both the disialoganglioside GD_2_, positive in 83% of patients, and B7-H3 protein, expressed in all the analyzed patients. This lower expression of NCL can be explained considering that cell surface NCL, whose translocation from the nucleus to the cytoplasm and to the cell membrane depends on a vesicular secretory pathway that is endoplasmic reticulum-Golgi complex independent [27], does not have an hydrophobic trans-membrane domain for anchorage in the plasma membrane [28]. Therefore, the presence and the degree of NCL expression on the external surface of tumor cells is constantly changing. Consequently, the non-positivity of some patients for cell surface NCL could depend on the fact that its expression evaluation was performed when the translocation to the membrane was not occur yet, rather than on a patient’s constitutional lack of NCL expression. Of note, a low NB infiltration seems to correspond to either low or absent NCL expression, while NCL positivity results higher and closely correlates with the expression of the other NB markers, when BM infiltration is more consistent.

NCL is one of the most abundant proteins in the nucleolus, where it mainly controls RNA metabolism and the biogenesis, assembly and maturation of ribosomes [4, 5]. An altered expression and function of NCL is implicated in various pathological processes, particularly in viral infections and tumorigenesis. For these reasons, NCL represents a potential target for the development of anti-viral and anti-tumor strategies [4, 5]. Different stimuli can alter the cellular distribution of NCL and, consequently, modify its function. Notably, both the cellular over-expression and its localization at the cell membrane level are peculiar characteristics of tumor cells and tumor endothelial cells [5]. Indeed, NCL can also be found in the nucleus, cytoplasm and cell membrane and its different pattern of localization reflects a different role in tumor development and growth. On the cell membrane, NCL binds Fas (apoptosis pathway), blocking cell death [29]. It interacts with Erb1 and Ras GDP and Ras GTP, increasing tumor development and proliferation, migration and resistance to apoptosis via MAPK/AKT pathways [30, 31]. NCL interacts with HGF, VEGF and TNF alpha inducing protein [4]. Moreover, cell surface NCL participates in the translocation of extracellular molecules, such as angiogenic factors, promoting angiogenesis [5]. Altogether, these are all valid reasons to justify the development of anti-tumor strategies based on cell surface NCL targeting. For instance, cell surface NCL play a pivotal role as the targeted receptor in the AS1411 aptamer-based therapy, whose therapeutic efficacy is demonstrated also in a clinical setting [32]. Cell surface NCL can be also targeted by pseudopeptides and antibodies. The NCL antagonists HB-19 and its analog N6L are able to impair tumor growth and tumor angiogenesis, to inhibit tumor progression and metastasization and to promote the normalization of tumor vasculature, features reported also after the administration of the anti-NCL antibody NCL3 [21, 33–35].

In our work, cell surface NCL was used as an internalizing target ligand to allow nanotechnology-based chemotherapy uptake from NB cells, leading to strong cytotoxic effects both in vitro and in vivo. On this scenario, an interesting point of discussion would be precisely represented by the use of these drug-loaded nanoparticles, decorated with the F3 peptide able to recognize NCL-expressing tumor cells. Indeed, in addition of being an efficient drug delivery system, the specific targeting of cell surface NCL, and/or the transport and internalization of the encapsulated cytotoxic drug into the tumor cells, might themselves cause a change in the cellular distribution of the NCL and, in particular, lead to a modification/down modulation of the NCL at the cell surface. Similarly, monitoring of cell surface NCL during and after chemotherapy treatment would be essential before suggesting its use in the clinical setting. These points, together with the identification of an optimal therapeutic window, need further studies. However, implantations of NCL-positive tumor fragments from NB patients into immuno-deficient mice (Patient Derived Xenografts-PDX) reveal that cell surface expression of NCL is maintained through the murine generations (Figure 8S). As a consequence, in the near future this will allow to have even more clinically relevant, NCL-expressing human NB cells in animal models, available for the development of precision medicine strategies [36, 37]. Finally, in the era of immuno-oncology, the recent implementation of the anti-GD_2_ antibody Dinutuximab into the standard of care has certainly improved high-risk NB patient outcomes, but 5-year survival rates are still below 50% [38]. In view of the results herein obtained, the simultaneous combination targeting of GD_2_, B7-H3 and NCL, by means of Dinutuximab [39], chimeric antigen receptor (CAR) T cells targeting B7-H3 (B7-H3.CAR-Ts) [40] and PEGASEMP™, might represent an innovative and useful strategy for refractory/relapsed NB patients.

## Conclusions

In this manuscript, cell surface Nucleolin (NCL) is proposed as an innovative cellular target for neuroblastoma (NB) therapies in pre-clinical and future clinical setting. NB, the most common solid tumor of infancy, is a pediatric cancer characterized by a wide clinical behavior and adverse outcome despite aggressive therapies. Since the use of targeted therapies is severely limited by the lack of specific receptors, chemotherapy and radiotherapy remain the main treatment choices for high-risk NB patients, which expose them to remarkable toxicity and to drug resistance development. Nanoparticles-mediated targeted therapies may improve efficacy and decrease toxicity. NCL is overexpressed and partially localized on the cell surface of tumor cells of adult cancers. On the other hand, at present nothing has been reported on cell surface NCL and pediatric tumors, and in particular on NB. In this work, the evaluation and the validation of cell surface NCL expression on tumor cell lines and particularly on primary NB cells, including patient-derived and Patient-Derived Xenografts-PDX, at the level of both tumor masses and tumor cells infiltrating the bone marrow (characteristic of disseminated high risk NB, difficult to eradicate) can be extremely important from both translational and clinical points of view. Moreover the anti-tumor effects obtained here by NCL-recognizing nanoparticles in vitro and in pre-clinical models of human NB represent the proof-of-concept supporting cell surface NCL as a potentially druggable cellular marker for NB patients.

## Supplementary Information

**Additional file 1: Supplementary Table 1.** Patient Codes of Bone Marrow (BM)*-infiltrating NB cells*

**Additional file 2: Figure 1S.** Representative images of single HTLA-230 and SH-SY5Y cells, stained with anti-NCL moAb and counted by Imaging Flow Cytometry. BF: Bright Field; 2^nd^ Ab: AlexaFluor 488-conjugated secondary moAb; NCL: anti-NCL AlexaFluor488 moAb (green).

**Additional file 3: Figure 2S.** Immunohistochemistry staining on formalin-fixed sections derived from healthy murine kidney and adrenal gland. Bar: 100 μm. Brown: NCL staining.

**Additional file 4: Figure 3S.** Representative pictures of single SH-SY5Y cells incubated with anti-NCL-A488 antibody (green) and rhodamine (Rhoda)-labeled, non-targeted liposomes (NT-Rhoda) (red), and analyzed by Imaging Flow Cytometry. BF: Bright Field; NCL: anti-NCL AlexaFluor488 moAb; 2^nd^ Ab: AlexaFluor 488-conjugated secondary moAb; NT-Rhoda: liposome; Combo: co-localization.

**Additional file 5: Figure 4S.** In vitro **effects of T-DXR on NB cell proliferation and viability*.*** **A)** CFSE assay. IMR-32 and NXS2 NB cell lines were treated with 1 μM of T-DXR. At 96 h after treatment, cells were collected and processed by FC to detect CFSE fluorescence. Results are expressed as MFI. Columns: MFI ± S.D. *, *p* < 0.05, T-DXR *vs* CTR; ***, *p* < 0.001, T-DXR *vs* CTR. **B)** Viability assay: NB (IMR-32, SH-SY5Y and NXS2) and skin keratinocytes (HaCaT) cells were treated with 0.5 μM DXR of T-DXR and processed to determine cytotoxicity through the MTS assay. Results are expressed as optical density (OD) determined at 490 nm. Columns: OD ± S.D. *, *p* < 0.05, T-DXR *vs* CTR; **, *p* < 0.01, T-DXR *vs* CTR; ***, *p* < 0.001, T-DXR *vs* CTR.

**Additional file 6: Figure 5S.** Mean body weight after F3 peptide-targeted, doxorubicin (DXR)-loaded, pH-sensitive nanoparticles treatment. Mice were injected in the adrenal gland with 1 x 10^6^ luciferase-transfected IMR-32 (IMR-32-luc) NB cells and treated as reported in Fig. 5 caption.

**Additional file 7: Figure 6S.** Hematological toxicity evaluation. Levels of red blood cells (RBC), hemoglobin (HGB), hematocrit (HCT), reticulocytes (RET), white blood cells (WBC) and platelets (PLT) were quantified in IMR-32-luc-bearing mice treated as reported in Fig. 5 caption.

**Additional file 8: Figure 7S.** Non hematological toxicity evaluation. Levels of serum albumin (ALB), phosphatase alkaline (ALP), glutamic-pyruvic transaminase (ALT), glutamic oxaloacetic transaminase (AST), lactate dehydrogenase (LDH), creatine phosphokinase (CK) creatinine (CREA) and Urea were quantified in IMR-32-luc-bearing mice treated as reported in Fig. 5 caption.

**Additional file 9: Figure 8S.** Cell surface NCL expression on tumor specimens from NB patients (left) and on Patient-Derived Xenografts (PDX) from the same patient (right). Tumor fragments from patients (Patient codes 4654 and 4736) were mechanically dissociated to single cells suspension, stained with anti-CD56-APC (CD56 APC) and anti-NCL-A488 (NCL FITC) moAbs and evaluated by Flow Cytometry. CD56: marker of NB cells. PDX-P2: 2^nd^ generation of PDX. The % of CD45-/CD56+/NCL+ cells are reported.

## Data Availability

Not applicable.

## Abbreviations

NB: Neuroblastoma
NCL: Nucleolin
DXR: doxorubicin
NT-DXR: Untargeted, DXR-loaded pH-sensitive liposomes
T-DXR: F3 peptide-targeted
DXR: loaded pH-sensitive liposomes.
