# Supplementary material for: Cell surface Nucleolin represents a novel cellular target for neuroblastoma therapy
Source: J Exp Clin Cancer Res. 2021 Jun 2;40:180. doi: 10.1186/s13046-021-01993-9 (PMC8170797; doi:10.1186/s13046-021-01993-9)
Supplement: Supplementary file 1 — Additional file 1: Supplementary Table 1. Patient Codes of Bone Marrow (BM)-infiltrating NB cells [file 13046_2021_1993_MOESM1_ESM.docx]

| Samples | Patient Code | Stage |
| --- | --- | --- |
| BM #1 | 3372 | M (relapse) |
| BM #2 | 4210 | M (relapse) |
| BM #3 | 4353 | M (relapse) |
| BM #4 | 4725 | M (relapse) |
| BM #5 | 4729 | M (relapse) |
| BM #6 | 4762 | M (relapse) |
| BM #7 | 4876 | M (relapse) |
|  |  |  |
| BM#1 | 4816 | M (onset) |
| BM#2 | 4826 | M (onset) |
| BM#3 | 4827 | M (onset) |
| BM#4 | 4852 | M (onset) |
| BM#5 | 4865 | M (onset) |
| BM#6 | 4873 | M (onset) |
| BM #7 | 4877 | M (onset) |
| BM #8 | 4891 | M (onset) |
| BM #9 | 4894 | M (onset) |
| BM #10 | 4895 | M (onset) |
| BM #11 | 4899 | M (onset) |
| BM #12 | 4904 | M (onset) |
| BM #13 | 4914 | M (onset) |
| BM #14 | 4922 | M (onset) |
| BM #15 | 4923 | M (onset) |
| BM #16 | 4930 | M (onset) |
| BM #17 | 4937 | M (onset) |
| BM #18 | 4942 | M (onset) |
| BM #19 | 4944 | M (onset) |
| BM #20 | 4978 | M (onset) |
| BM #21 | 5000 | M (onset) |
| BM #22 | 5004 | M (onset) |
| BM #23 | 5005 | M (onset) |
| BM #24 | 5010 | M (onset) |
| BM #25 | 5012 | M (onset) |
| BM #26 | 5013 | M (onset) |
| BM #27 | 5014 | M (onset) |
| BM #28 | 5018 | M (onset) |
| BM #29 | 5021 | M (onset) |

**Supplementary Table 1:** Patient Codes of Bone Marrow (BM)-infiltrating NB cells
