# Supplementary figures and images for: Cell surface Nucleolin represents a novel cellular target for neuroblastoma therapy
Source: J Exp Clin Cancer Res. 2021 Jun 2;40:180. doi: 10.1186/s13046-021-01993-9 (PMC8170797; doi:10.1186/s13046-021-01993-9)

## Slide 1
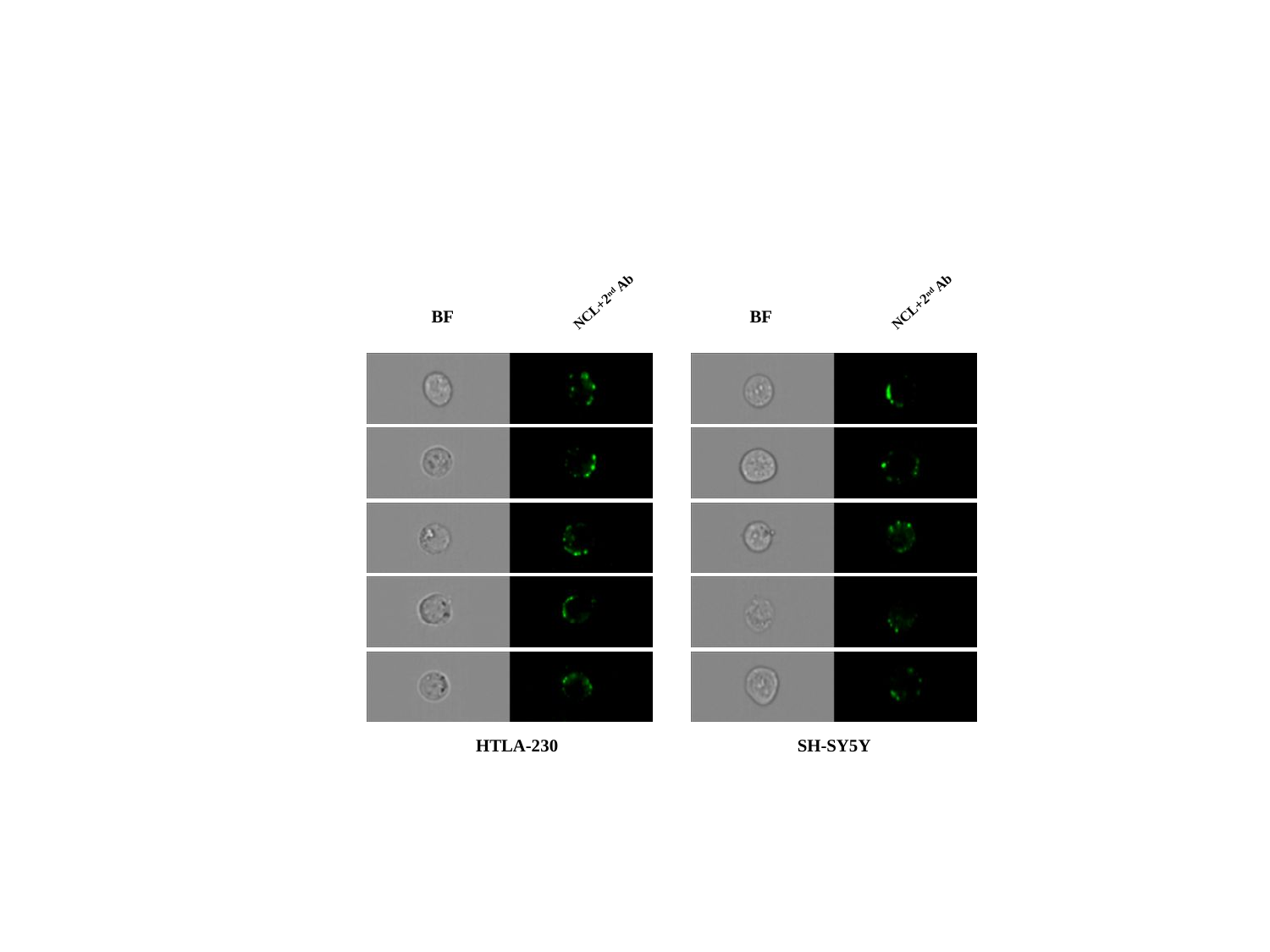

NCL+2nd Ab
NCL+2nd Ab
BF
BF
HTLA-230
SH-SY5Y

Supplement: Supplementary file 2 — Additional file 2: Figure 1S. Representative images of single HTLA-230 and SH-SY5Y cells, stained with anti-NCL moAb and counted by Imaging Flow Cytometry. BF: Bright Field; 2nd Ab: AlexaFluor 488-conjugated secondary moAb; NCL: anti-NCL AlexaFluor488 moAb (green). [file 13046_2021_1993_MOESM2_ESM.pptx]

## Slide 1
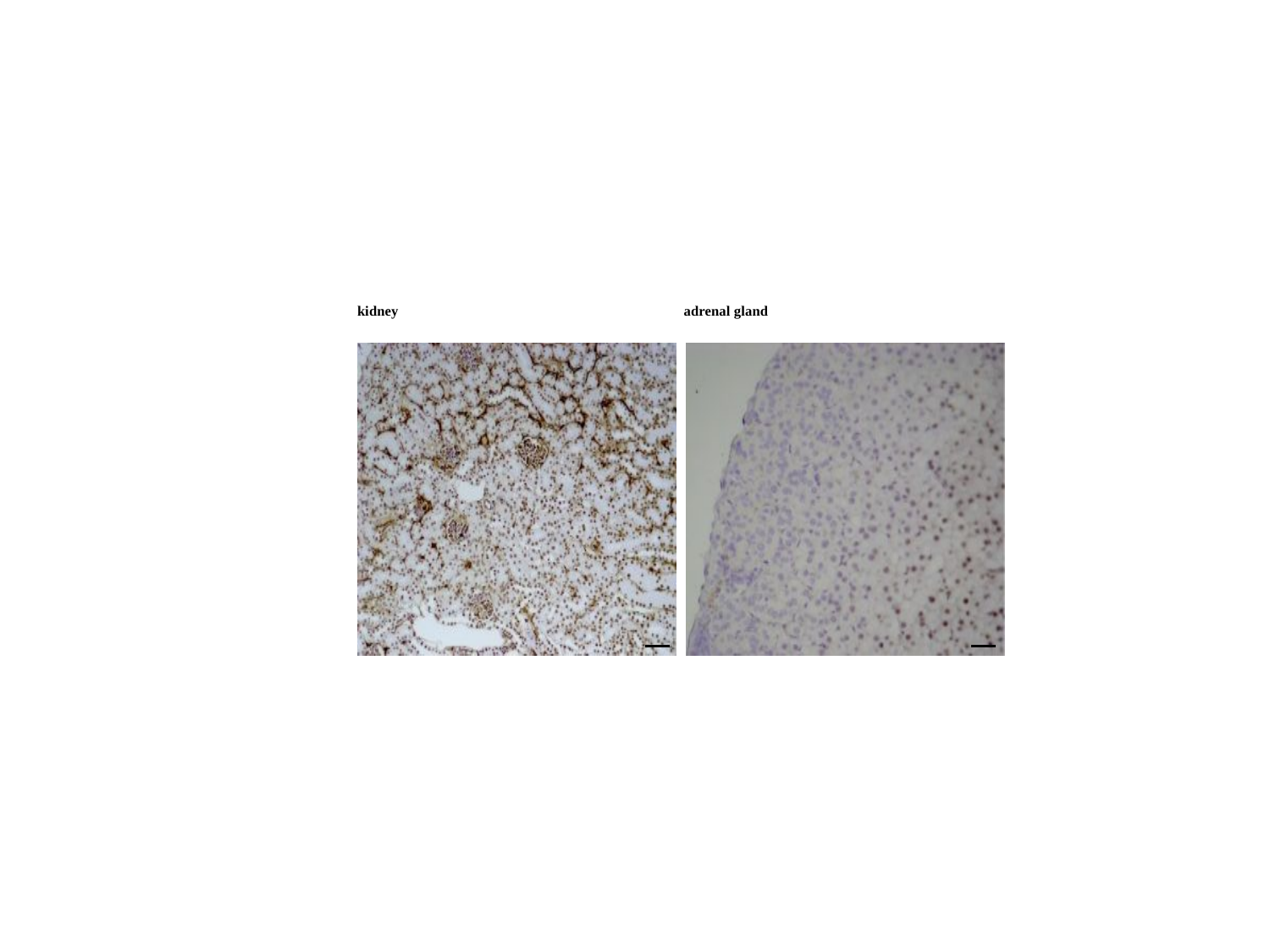

kidney
adrenal gland

Supplement: Supplementary file 3 — Additional file 3: Figure 2S. Immunohistochemistry staining on formalin-fixed sections derived from healthy murine kidney and adrenal gland. Bar: 100 μm. Brown: NCL staining. [file 13046_2021_1993_MOESM3_ESM.pptx]

## Slide 1
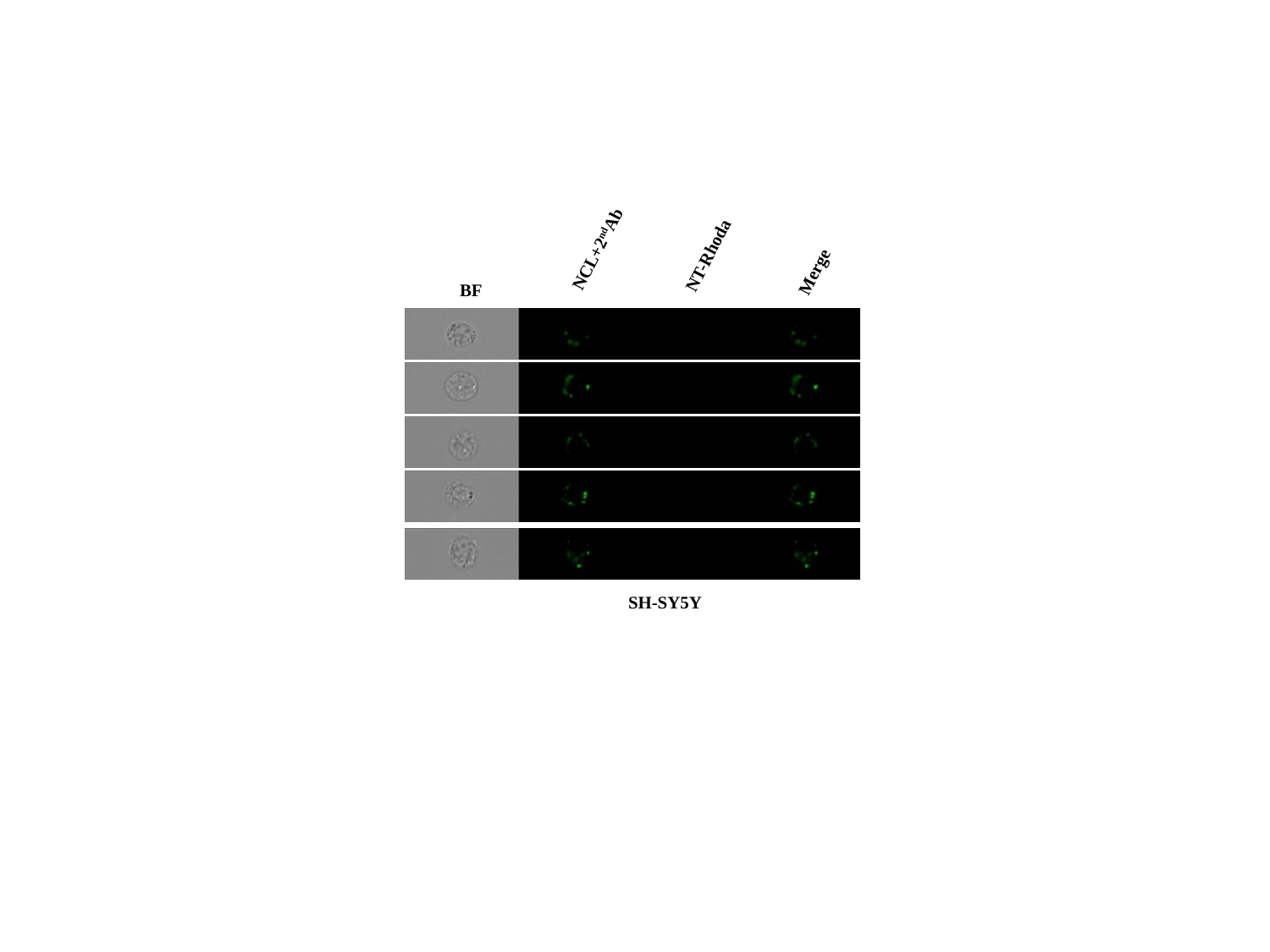

NCL+2ndAb
NT-Rhoda
Merge
BF
SH-SY5Y

Supplement: Supplementary file 4 — Additional file 4: Figure 3S. Representative pictures of single SH-SY5Y cells incubated with anti-NCL-A488 antibody (green) and rhodamine (Rhoda)-labeled, non-targeted liposomes (NT-Rhoda) (red), and analyzed by Imaging Flow Cytometry. BF: Bright Field; NCL: anti-NCL AlexaFluor488 moAb; 2nd Ab: AlexaFluor 488-conjugated secondary moAb; NT-Rhoda: liposome; Combo: co-localization. [file 13046_2021_1993_MOESM4_ESM.pptx]

## Slide 1
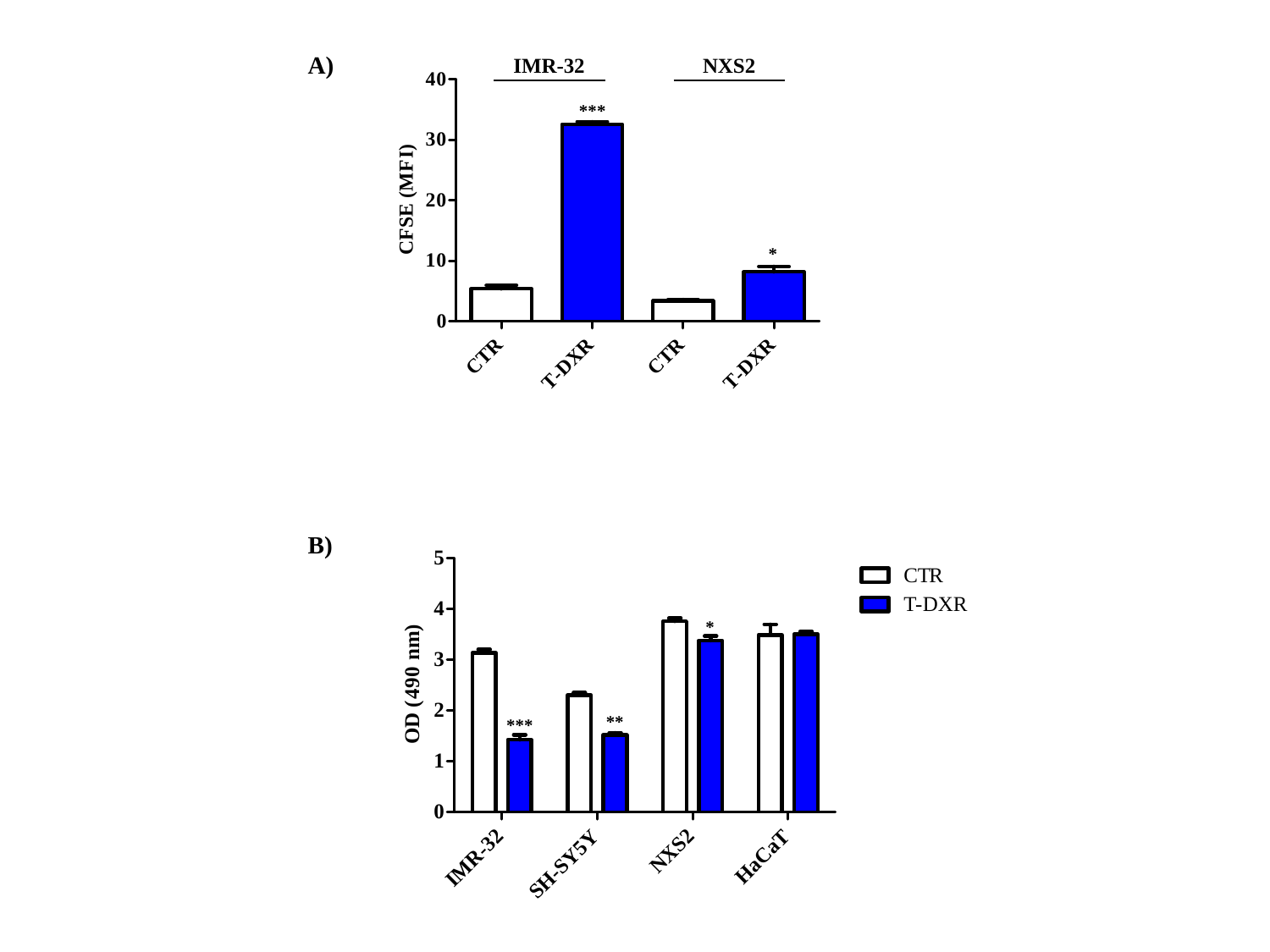

A)
***
*
IMR-32
NXS2
*
**
***
B)

Supplement: Supplementary file 5 — Additional file 5: Figure 4S. In vitro effects of T-DXR on NB cell proliferation and viability. A) CFSE assay. IMR-32 and NXS2 NB cell lines were treated with 1 μM of T-DXR. At 96 h after treatment, cells were collected and processed by FC to detect CFSE fluorescence. Results are expressed as MFI. Columns: MFI ± S.D. *, p < 0.05, T-DXR vs CTR; ***, p < 0.001, T-DXR vs CTR. B) Viability assay: NB (IMR-32, SH-SY5Y and NXS2) and skin keratinocytes (HaCaT) cells were treated with 0.5 μM DXR of T-DXR and processed to determine cytotoxicity through the MTS assay. Results are expressed as optical density (OD) determined at 490 nm. Columns: OD ± S.D. *, p < 0.05, T-DXR vs CTR; **, p < 0.01, T-DXR vs CTR; ***, p < 0.001, T-DXR vs CTR. [file 13046_2021_1993_MOESM5_ESM.pptx]

## Slide 1
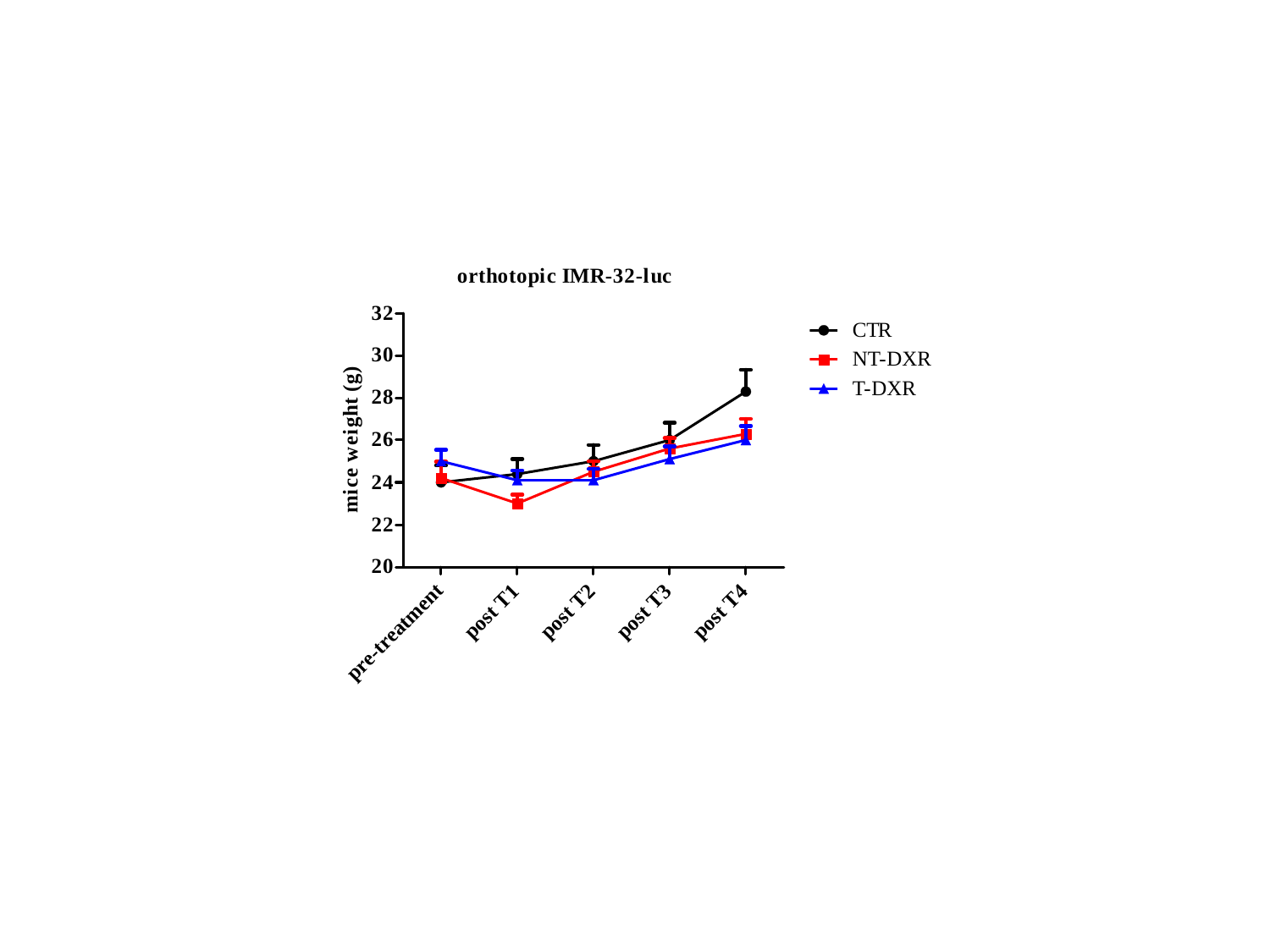

Supplement: Supplementary file 6 — Additional file 6: Figure 5S. Mean body weight after F3 peptide-targeted, doxorubicin (DXR)-loaded, pH-sensitive nanoparticles treatment. Mice were injected in the adrenal gland with 1 x 10^6^ luciferase-transfected IMR-32 (IMR-32-luc) NB cells and treated as reported in Fig. 5 caption. [file 13046_2021_1993_MOESM6_ESM.pptx]

## Slide 1
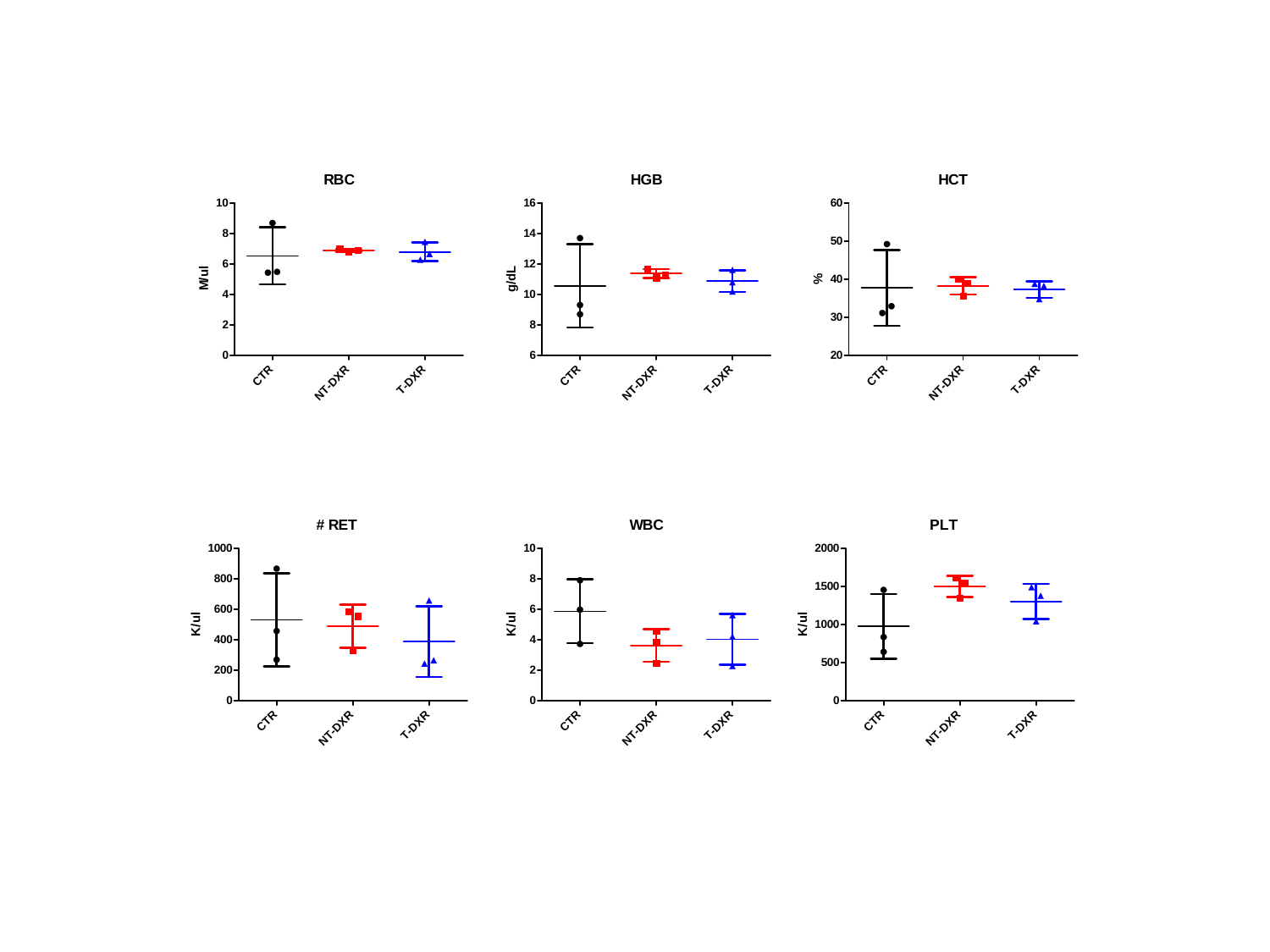

Supplement: Supplementary file 7 — Additional file 7: Figure 6S. Hematological toxicity evaluation. Levels of red blood cells (RBC), hemoglobin (HGB), hematocrit (HCT), reticulocytes (RET), white blood cells (WBC) and platelets (PLT) were quantified in IMR-32-luc-bearing mice treated as reported in Fig. 5 caption. [file 13046_2021_1993_MOESM7_ESM.pptx]

## Slide 1
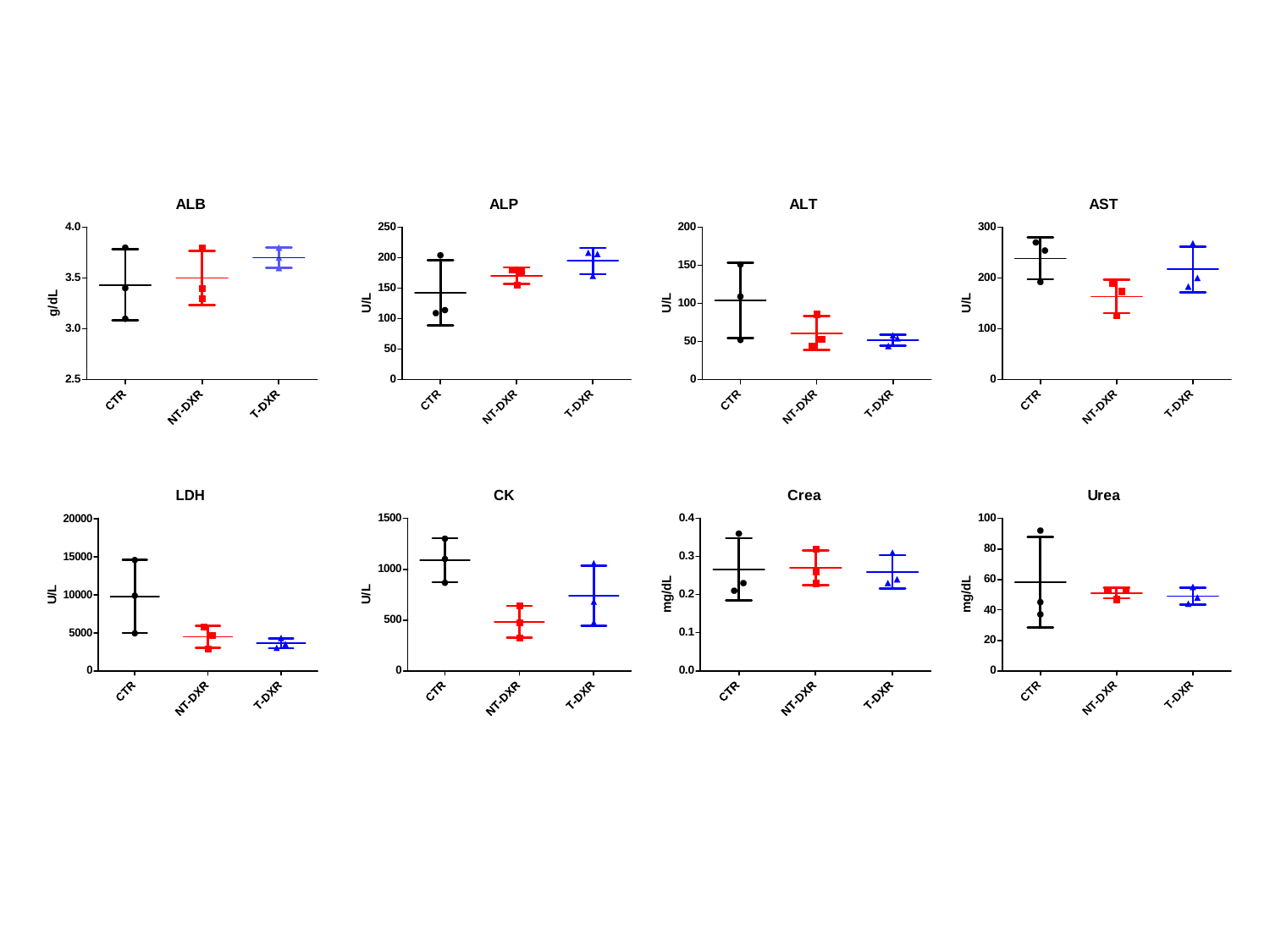

Supplement: Supplementary file 8 — Additional file 8: Figure 7S. Non hematological toxicity evaluation. Levels of serum albumin (ALB), phosphatase alkaline (ALP), glutamic-pyruvic transaminase (ALT), glutamic oxaloacetic transaminase (AST), lactate dehydrogenase (LDH), creatine phosphokinase (CK) creatinine (CREA) and Urea were quantified in IMR-32-luc-bearing mice treated as reported in Fig. 5 caption. [file 13046_2021_1993_MOESM8_ESM.pptx]

## Slide 1
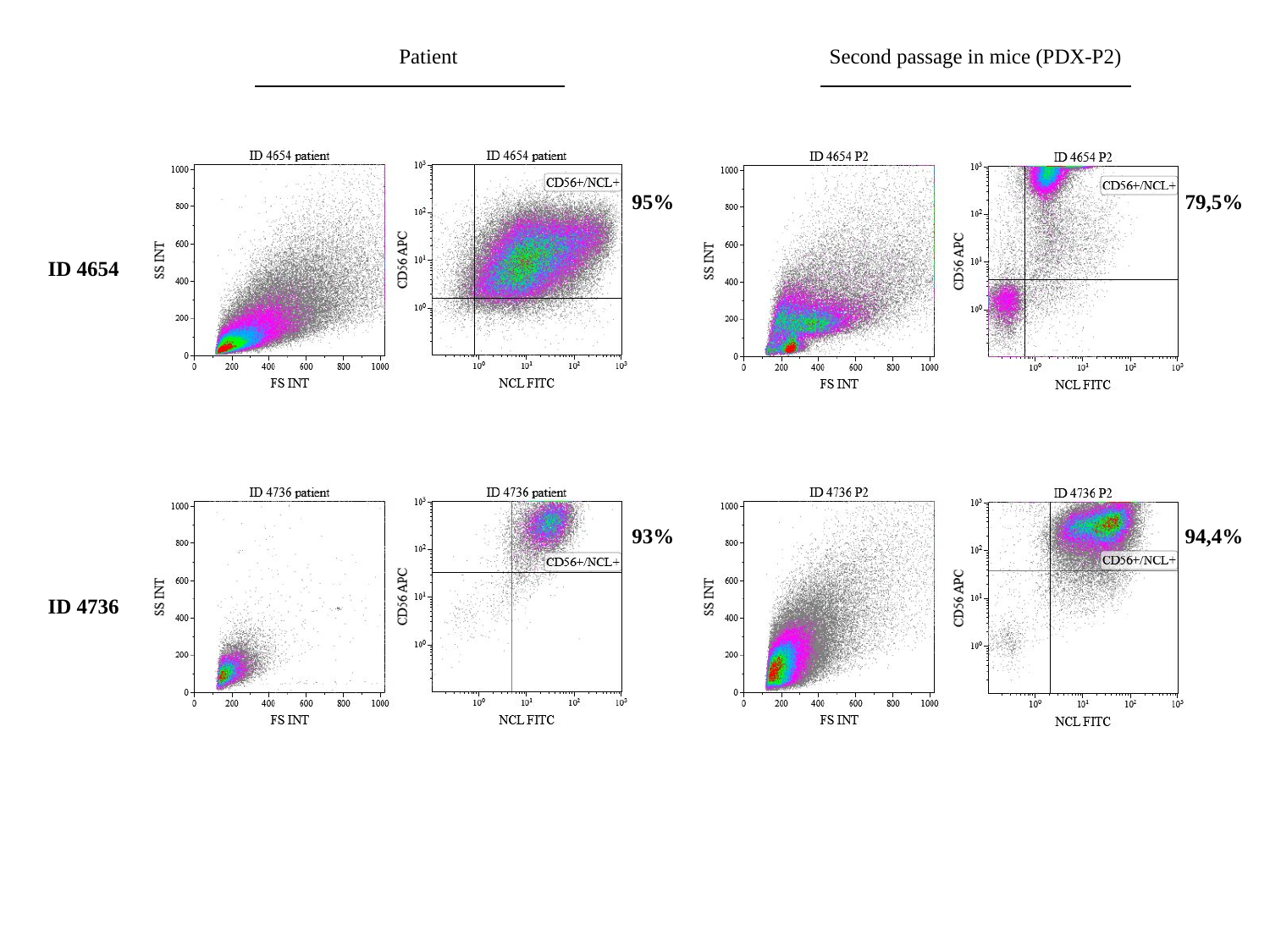

Patient
Second passage in mice (PDX-P2)
95%
79,5%
ID 4654
ID 4736
93%
94,4%

Supplement: Supplementary file 9 — Additional file 9: Figure 8S. Cell surface NCL expression on tumor specimens from NB patients (left) and on Patient-Derived Xenografts (PDX) from the same patient (right). Tumor fragments from patients (Patient codes 4654 and 4736) were mechanically dissociated to single cells suspension, stained with anti-CD56-APC (CD56 APC) and anti-NCL-A488 (NCL FITC) moAbs and evaluated by Flow Cytometry. CD56: marker of NB cells. PDX-P2: 2nd generation of PDX. The % of CD45-/CD56+/NCL+ cells are reported. [file 13046_2021_1993_MOESM9_ESM.pptx]
